# Giant trees enhance zoochorous plant dispersal in sacred groves and human settlements of the Western Ghats, India

**DOI:** 10.1371/journal.pone.0343978

**Published:** 2026-05-04

**Authors:** Omkar Pai, Jayant Sarnaik, Archana Godbole, Gunwant Mahajan, Akshay Gawade, Kevin Matteson

**Affiliations:** 1 Applied Environmental Research Foundation, Pune, India; 2 Project Dragonfly/Department of Biology, Miami University, Oxford, Ohio, United States of America; Universitas Airlangga, INDONESIA

## Abstract

In India and other countries, large and ancient trees that persist in sacred groves and human-dominated landscapes support ecosystem services, including frugivore-mediated seed dispersal. In this study, we assessed the ecological significance of ‘giant’ trees (>1 m trunk circumference) in Vanzole Village, Western Ghats, India. We documented 82 giant trees belonging to 20 species in four sacred groves and surrounding human settlements. Sacred groves harbored a higher species richness of large trees (14 species, N = 44) than human settlements (8 species, N = 38). Tree composition varied, with *Mangifera indica* (Mango) and *Artocarpus heterophyllus* (Jackfruit) dominating in human settlements, whereas *Terminalia bellirica* (Beheda) and several other species were more abundant in sacred groves. Giant trees in human settlements exhibited larger canopy diameter (p = 0.002) and DBH (p < 0.001), while trees in sacred groves were significantly taller (p < 0.001). Sapling recruitment was notably higher in sacred groves; *Caryota urens* saplings were found under 91% of trees in groves (mean = 56.0) versus 47% in settlements (mean = 4.9). The larger-seeded *Strychnos nux-vomica* was nearly absent in human settlements (5%) compared to sacred groves (77%). Our study highlights the unique conservation value of sacred groves due to their retention of numerous and diverse giant trees. Together with remnant large trees in human settlements, this network is vital for sustaining the regeneration of zoochorous species by supporting the activity of hornbills and other frugivores.

## Introduction

Giant trees or large diameter trees, also referred to as old-growth or emergent trees, are fundamental to the structure, dynamics, and functioning of both temperate and tropical forest ecosystems [1]. Ecologically, giant trees play a crucial role in carbon sequestration, storing substantial amounts of biomass and carbon [2] compared to smaller trees [3]. Giant trees can also regulate microclimates and influence forest regeneration and succession patterns [4], and may serve as individual ecosystems, hosting arboreal fauna and epiphytic plants. Many birds, such as Great Hornbills (*Buceros bicornis*), nest in the cavities of giant (often emergent) trees in protected forests [5], and other forest-dependent birds are known to benefit from large trees with connected canopies [6].

Remnant giant trees in villages of the Northern Western Ghats are sometimes protected by local communities for their cultural value or food provisioning; while larger populations of giant trees are also conserved within protected habitats such as sacred groves. Sacred groves are areas of primary forest [7] that are relatively undisturbed and protected by local communities due to their spiritual significance, often associated with local deities or ancestral worship [8]. While the ecological value of giant trees is generally understood, their importance within sacred groves and as remnant trees in human settlements is still underexplored. Specifically, it is unclear to what degree these trees act as stepping stones [9] or corridor nodes [10] for birds, supporting activities like nesting and roosting, and facilitating seed dispersal throughout human-altered landscapes.

There are over 100,000 sacred groves across India contributing to community-based conservation and providing a myriad of forest-based ecosystem services [11]. Local communities have deeply-held beliefs that protect sacred groves from exploitation, which helps maintain their biodiversity [7,12,13]. Specifically, cultural practices such as taboos against cutting trees contribute to the preservation of flora and fauna, including birds [14]. Researchers have conducted inventories of the plant diversity of sacred groves [15–17], noting the presence of endemic and rare species [18], medicinal and other culturally-valued plants [19,20], and overall diversity comparable to surrounding semi-natural areas [21,22].

Sacred groves have also been shown to support biodiversity comparable to larger and less-disturbed ecosystems. Jyothi & Nameer [23] found a total of 111 bird species, including numerous canopy-feeding birds, within 15 sacred groves in Kerala, India. Joshi and Sarnaik [10] found a similar high diversity of 101 bird species, with many “middle canopy nesters,” in 19 sacred groves of Sangameshwar Taluka, India. Bhagwat et al. [21] found that the diversity of trees, birds, and fungi in sacred groves was comparable to that in nearby forests and coffee plantations. Similarly, Kumar et al. [11] identified 147 bird species in 60 groves in the Aravalli range, Rajasthan, India, and found bird diversity in sacred groves to be similar to two nearby reserves. Khan et al. [18] highlighted anecdotal observations of endemic species in sacred groves, such as climber (vine) species found nowhere else, northern range limits of certain plants, and the presence of unique species such as *Macaca silenus* (Lion-tailed Macaques). Prasad & Bharathi [24] documented 98 tree species of which 66 were regenerating via saplings in four sacred groves in the Western Ghats. Kulkarni et al. [25] found an impressive 296 species of plant, in 100 plant families, within 15 sacred groves in the Pune District of Maharashtra, India. These and other studies highlight the biodiversity conservation value of sacred groves.

Frugivorous birds, which feed on fruits and disperse seeds through their droppings, are critical for the regeneration of trees and other flora in Asia [26]. Their activities ensure the continuation of forest ecosystems by facilitating seed germination and plant growth. By attracting nesting or roosting frugivorous birds, large trees may facilitate seed dispersal from parent plants, reducing loss due to predation and increasing their chances of survival [27]. Both *Caryota urens* (Fish-tail palm) and *Strychnos nux-vomica* (Kajra) are adapted for frugivore-mediated seed dispersal by hornbills [28,29], other birds, and mammals*.* Hornbills are considered the most important group of seed dispersers for large-fruited (> 2 cm diameter) woody species [30], eating a diversity of fruits and distributing them over large distances [26]. A high density of large-seeded saplings below a non-parent plant suggests frequent use of the tree canopy by frugivorous species. Seeds accumulating under trees used for nesting or roosting by hornbills have been shown to germinate well despite their aggregation [31,32]. However, the behavior of frugivorous birds may differ in human-altered landscapes [33], for example, if they preferentially utilize taller trees or trees in less-disturbed habitats.

**Fig 1:**
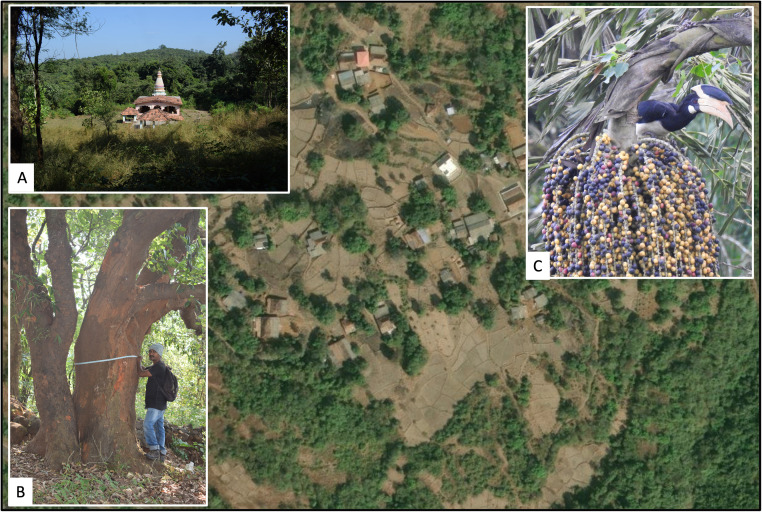
This study was conducted in Vanzole Village in the Northern Western Ghats of India. The landscape is a mix of human settlements, agriculture, forest, and sacred groves. We measured tree size (DBH, canopy, and height) and sapling recruitment under 82 trees in this area. Inset images: **A)** Sacred Grove of Kedarling; **B)** Team member, Sachin Parshram, measuring DBH of a giant tree; **C)** A Malabar Pied Hornbill (Anthracoceros coronatus) feeding on fruits of Caryota urens, one of the two focal tree species for which we measured sapling regeneration under giant trees in this study (Photo credit: Akshay Gawade).

We assessed the diversity of large-diameter trees (giant trees) and the degree of sapling recruitment under them, in several sacred groves and human settlement areas located in Vanzole Village in the Northern Western Ghats of India (Fig 1). This study addresses the following key questions: 1) How do the species richness and physical dimensions of giant trees differ between sacred groves and human settlements? 2) Does the recruitment of zoochorous saplings under giant trees differ between these two land use types? We hypothesized that giant trees in sacred groves would be larger and more diverse compared to giant trees embedded in settlements. We also expected higher recruitment of species such as *Caryota urens* and *Strychnos nux-vomica*, under trees located in sacred groves, relative to large trees in human settlements. This prediction is based on the understanding that greater zoochorous sapling recruitment beneath giant trees is an indicator of their use by frugivores, facilitating seed dispersal. Understanding patterns of frugivore-mediated sapling regeneration is particularly important in regions like the Western Ghats of India, where high levels of species endemism and biodiversity are threatened by invasive species, resource extraction, fire, livestock grazing, and other factors [34].

## Materials and methods

### Study sites

The Western Ghats is a mountain range that runs parallel to the western coast of peninsular India, spanning approximately 1,600 kilometers across six states: Gujarat, Maharashtra, Goa, Karnataka, Tamil Nadu, and Kerala. Covering an area of 140,000 square kilometers, this ecologically significant region [59] harbors some of the world’s most diverse and unique biodiversity [34,60].

Vanzole is a village in the Northern Western Ghats, adjacent to the Vanzole Ghat, with a total area of 988 hectares and a population of 1657 (https://censusindia.gov.in/census.website/). For administrative purposes, the village is divided into three parts—Vanzole, Vanzole Khurd, and Vanzole Budhruk—comprising 10 hamlets in total; two of these hamlets were included in the giant tree census. The village is located in a valley with fragmented agricultural lands, some of which are actively cultivated, primarily with rice, while others are abandoned and have given way to secondary forest growth. The hills around the valley include fragmented private forest.

There are four sacred groves in the village: the Sacred Grove of the Chandki Devi, the Sacred Grove of Dhanin Devi, and the Sacred Grove of Kedarling (all situated within private forest), and the Sacred Grove of Jugai Devi (located in one of the village hamlets surrounded by human settlements). The size of these sacred groves ranged from 2307 m^2^ up to 14650 m^2,^ with an average of 6587 m^2^.

The Western Ghats are home to four species of sympatric hornbills: Great Hornbill (*Buceros bicornis*), the Malabar Pied Hornbill (*Anthracoceros coronatus*), the Indian Grey Hornbill (*Ocyceros birostris*), and the Malabar Grey Hornbill (*Ocyceros griseus*) [55]. Other relatively common frugivorous birds and mammals include Nilgiri Wood Pigeon (*Columba elphinstonii*), Giant Squirrel (*Ratufa indica*), and some fruit bats. These frugivorous species are known to frequent the forest patches and large trees found within the study area.

No permits were required for this work, as the human settlements and sacred groves in Vanzole village are open access areas, and no oversight is required for research conducted on plants in this region.

### Locating and measuring giant trees

In addition to our ecological questions, this project aimed to document the giant trees of Vanzole village, where communities have traditionally protected sacred groves and individual giant trees in private forests and around human settlements. We worked with local knowledge holders from Vanzole Village to locate the sacred groves and giant trees in human settlements. We sampled ‘giant trees’, those with >1 m trunk circumference. Trees were identified to species by authors GM and OP using the website Flowers of India (https://www.flowersofindia.net/) and Ingalharikar [61]. For each tree, we measured the following three physical parameters: Diameter at Breast Height (DBH), tree height, and canopy diameter. To determine the canopy diameter, the furthest tip (i.e., the longest branch) from the trunk was located and the distance from it to exactly opposite end of the canopy across the trunk was measured. Tree height was estimated by both observers using a reference point and visual aids. All giant trees within the boundaries of each sacred grove were recorded.

### Assessing sapling recruitment

*Caryota urens* (Fish-tail palm; Arecaceae) and *Strychnos nux-vomica* (Kajra; Loganiaceae) are important tree species whose fruits are consumed and dispersed by frugivorous birds. We focused on these two species as indicators of frugivore-mediated dispersal for several reasons. First, both species are readily identifiable as saplings, with *C. urens* displaying pinnate leaves in its juvenile stage before developing its characteristic bipinnate, fish-tail shaped leaves [62]. *S. nux-vomica* saplings are recognized by their deep green, shiny, opposite leaves and smooth ashen bark [63]. Second, unlike some other fruit-bearing trees such as *Ficus*, which are consumed and dispersed by a broad spectrum of taxa, including smaller species and mammals [26], both *C. urens* and *S. nux-vomica* are primarily utilized by a more restricted subset of frugivorous species due to the substantial size of their seeds. The fruits of *C. urens* are small drupes measuring approximately 1 cm in diameter [64]. Members of our research team have observed species such as Malabar pied hornbill (*Anthracoceros coronatus*), Nilgiri wood pigeon (*Columba elphistonii*), and Asian koel (*Eudynamys scolopaceus*) feeding on *Caryota urens* seeds. The larger fruits of *Strychnos nux-vomica* (5–6 cm diameter) are toxic to many vertebrates due to the presence of strychnine and brucine, and are more exclusively consumed and dispersed by hornbills [28], particularly Malabar pied hornbills, which may have a tolerance for these toxins. Finally, *C. urens* and *S. nux-vomica* were relatively scarce as giant trees in this area. Therefore, the presence of saplings of these two species beneath larger, heterospecific trees is a strong indicator of zoochory. Importantly, these two zoochorous species were relatively abundant and easily identifiable compared to other zoochorous species in the area, making them good indicators of frugivore presence and seed dispersal.

To investigate the role of giant trees in supporting frugivorous birds, we assessed the presence of *C. urens* and *S. nux-vomica* saplings under sampled trees. We established 10-meter radius quadrats centered on the trunk of each tree and counted all saplings within these areas. Unlike many other regions, the understory vegetation in the human settlements of our study site is generally not actively managed through mowing, weeding, or biocide application. However, the presence of cattle introduces the potential for grazing, and both humans and cattle may contribute to trampling of young vegetation in heavily trafficked areas. Consequently, while the presence of saplings beneath heterospecific trees can indicate frugivore-mediated seed dispersal and subsequent recruitment, the absence of saplings in human settlements may reflect regeneration failure due to other factors, not necessarily the absence of frugivorous dispersal.

### Statistical analysis

We evaluated correlations among tree DBH, canopy diameter, and tree height. Because the data were not normally distributed (Shapiro-Wilks <0.05), we used Spearman’s correlations to evaluate these relationships.

We compared the species richness and composition of trees in sacred groves and human settlements. To address Question 1, we evaluated differences in tree size between sacred groves and human settlements. Specifically, we used Mann-Whitney tests to compare DBH, canopy diameter, and tree height across both land use types. To address Question 2 on differences in sapling recruitment between sacred groves and human settlements, we used Mann-Whitney tests to evaluate differences in *C. urens* and *S. nux-vomica* sapling recruitment beneath sampled giant trees in both location types.

## Results

### Biodiversity of giant trees

We documented a total of 82 giant trees, representing 20 species, across four sacred groves and two human settlement areas (hamlets) in Vanzole village, in the Northern Western Ghats. There were 14 species of giant tree in the sacred groves (N = 44) and eight species of giant tree in the human settlements (N = 38) (Table 1).

**Table 1:** Giant trees identified in human settlements and sacred groves of Vanzole Village in the Western Ghats from August 2018 to May 2019. Note that this is a list of large trees only (> 1m circumference), not a comprehensive list of all tree species in the area. In addition to the species listed below, three trees in sacred groves were not identified to the species level. We include select human uses and ecological values based on the Flowers of India website (https://www.flowersofindia.net/), sources therein, and individual citations noted.

Scientific Name	English/ Local Name(s)	Family	Select Human and Ecological Values	N (% of total)	# (%) in Human Settlements	# (%) in Sacred Groves
*Mangifera indica*	Mango/ Amba	Anacardiaceae	Fruit is consumed in ripe and unripe form [35]; Pollination by three species of honey bee (*Apis dorsata*, *A. cerana*, and *A. florea*) that visit the flowers consistently [36].	22 (27%)	19 (86%)	3 (14%)
*Terminalia bellirica*	Beheda/ Bhibitaki	Combretaceae	Fruit is used in Ayurvedic medicine, Triphala churna, as a laxative and improve digestion.	14 (17%)		14 (100%)
*Artocarpus heterophyllus*	Jackfruit/ Fanas	Moraceae	Fruit is edible; Pulp used to make sweets; Seeds have diuretic properties and are used in Ayurvedic medicine [37].	11 (13%)	11 (100%)	
*Flacourtia montana*	Atkin	Salicaceae	Endemic to the Western Ghats; Larval host plant of butterflies [38].	5 (6%)		5 (100%)
*Sizzygium cumini*	Malabar Plum/ Black Plum/ Jambhul	Myrtaceae	Fruit is edible; Fruits and seeds show hypoglycemic effects [39].	5 (6%)		5 (100%)
*Lagerstroemia microcarpa*	Nanya	Lythraceae	Endemic to the Western Ghats; The wood has high timber value [40].	3 (4%)		3 (100%)
*Bombax ceiba*	Katesawar	Malvaceae	The wood is traditionally used for preparing plywood, canoes, light-duty boats, etc. (https://www.flowersofindia.net/)	2 (2%)		2 (100%)
*Ficus amplissima*	Payar	Moraceae	Fruit is an important food source for various frugivores [41].	2 (2%)	1 (50%)	1 (50%)
*Ficus benghalensis*	Banyan Tree/ Vad	Moraceae	Tree has cultural/spiritual importance; Fruits are an important food source for various frugivores [42].	2 (2%)	2 (100%)	
*Strychnos nux-vomica*	Kajra	Loganiacea	Medicinal properties; Used to treat digestive issues and as a general tonic (https://www.flowersofindia.net/).	2 (2%)		2 (100%)
*Terminalia elliptica*	Ain	Combretaceae	Tree has high timber value [43].	2 (2%)	2 (100%)	
*Careya arborea*	Kumbha	Lecythidaceae	Local communities crush the fruits and use them as fertilizer (O. Pai, personal communication).	1 (1%)		1 (100%)
*Caryota urens*	Fish Tail Palm/ Bherlimaad	Arecaceae	Fruits are an important food source for various frugivores [44].	1 (1%)	1 (100%)	
*Dillenia pentagyna*	Karambel	Dilleniaceae	Medicinal properties used by local communities [45].	1 (1%)		1 (100%)
*Garuga pinnata*	Kakad	Burseraceae	Medicinal properties: Paste of leaves used to treat headache and fever [46].	1 (1%)		1 (100%)
*Melia dubia*	Nimbera	Meliaceae	Leaf litterfall and nutrient recycling enhance soil health, contributing to sustainable agriculture [47].	1 (1%)	1 (100%)	
*Mimusops elengi*	Bakul	Sapotaceae	Important plant in the Ayurvedic system of medicine: traditionally used as an astringent and tonic against stomach-related ailments [48].	1 (1%)		1 (100%)
*Tectona grandis*	Teak/ Saag	Lamiaceae	Highly valued for good quality timber [49], used for the construction of houses and furniture.	1 (1%)		1 (100%)
*Terminalia chebula*	Hirda/ Haritaki	Combretaceae	Fruits traditionally used in Ayurvedic medicine, Triphala churna [50], as a laxative and to improve digestion.	1 (1%)	1 (100%)	
*Xantolis tomentosa*	Katekumbal	Sapotaceae	Used by local communities to treat chronic joint pain [51].	1 (1%)		1 (100%)
Total species (N)				20 (82)	8 (38)	14 (44)

### Giant trees in sacred groves and human settlements

Six tree species were only observed in the human settlements, whereas 12 species were only observed in the sacred groves. Only two tree species were found in both sacred groves and human settlements. Species exclusively found in human settlements included several trees that provide food resources, such as *Artocarpus heterophyllus* (Jackfruit) and *Mangifera indica* (Mango), or have medicinal, lumber, or spiritual value, such as *Terminalia chedbula* (Hirda/Haritaki/Myrobalan), *Terminalia elliptica* (Indian Laurel), *Melia dubia* (Nimbera), and *Ficus benghalensis* (Banyan). Species only observed in the sacred groves as giant trees included *Lagerstroemia microcarpa* (Nanya), *Syzygium cumini* (Malabar Plum), *Tectona grandis* (Teak), *Careya arborea* (Kumbha), *Mimusops elengi* (Bakul), *Flacourtia montana* (Mountain sweet thorn), *Terminalia bellirica (Bheheda)*, *Strychnos nux-vomica* (Kajra), *Bombax ceiba* (Katesavar), *Dillenia pentagyna* (Karambel), *Xantolis tomentosa* (Katekumbal), and *Garuga pinnata* (Kakad).

The highest occurrence of large diameter trees of *T. bellirica* (Beheda) was recorded in sacred groves, with a total of 14 individuals identified. Of these, the Sacred Grove of Jugai Devi hosted the largest number, with seven giant *T. bellirica* trees, followed by the Sacred Grove of Kedaling with five, and the Sacred Grove of Dhanin Devi with two.

*Mangifera indica* (Mango) and *Artocarpus heterophyllus* (Jackfruit) were the most frequently documented species in human settlements. 19 of 22 (86%) of the *M. indica* trees were observed in human settlements, and all 11 *A. heterophyllus* were found exclusively in human settlements. The fruits of these species are commonly consumed by local inhabitants.

### DBH (Diameter at Breast Height), canopy, and height of giant trees

**Fig 2:**
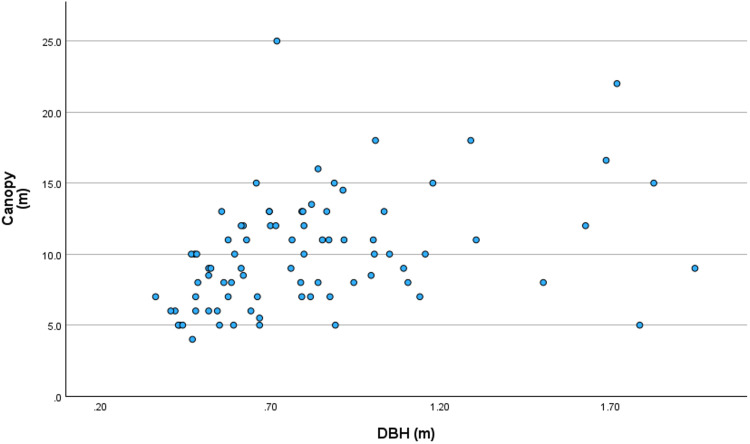
Relationship between tree DBH (m) and canopy diameter (m). The scattered plot represents the relationship between DBH and the canopy diameter of 82 giant trees located in human settlements and sacred groves of Vanzole village, in the Northern Western Ghats.

Tree DBH and canopy diameter were highly correlated (Spearman’s r = 0.452, p < 0.001; Fig 2). Also significant, but with a weaker relationship, was the correlation between tree height and tree DBH (Spearman’s r = 0.255, p = 0.021) and tree height and canopy diameter (Spearman’s r = 0.238, p = 0.032).

**Fig 3:**
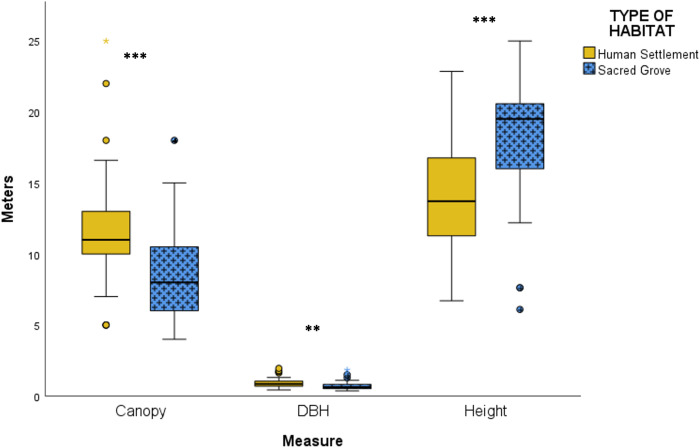
Comparison among canopy diameter, tree DBH, and tree height differences. The median and one quartile box plots compare canopy diameter, tree DBH, and tree height differences of 82 giant trees measured in human settlements and sacred groves of Vanzole village, in the Northern Western Ghats. The box represents the interquartile range (IQR) with the center line denoting the median. Whiskers extend to the highest and lowest values within 1.5 times IQR. Open circles indicate mild outliers (1.5 times IQR to 3 times IQR), stars indicate extreme outliers (>3 times IQR) and asterisks indicate significance (*** p < 0.001; ** p < 0.01, * < 0.05).

Compared to sacred groves, giant trees in human settlements had significantly wider canopy diameter (Mann-Whitney U = 1171, p = 0.002) and DBH (Mann-Whitney U = 1211.5, p < 0.001). In contrast, tree height was significantly greater in the sacred groves than in human settlements (Mann-Whitney U = 465.5, p < 0.001) (Fig 3).

### Recruitment of zoochorous saplings in sacred groves and human settlements

At least one *C. urens* sapling was found under 71% of all sampled trees. In sacred groves, 91% of sampled trees had at least one *C. urens* sapling, whereas in human settlements, this frequency was 47%. There were significantly more saplings of *C. urens* under giant trees in sacred groves (mean = 56.0, median = 35, SD = 51.4) than under giant trees in human settlements (mean = 4.9, median = 0, SD = 10.5) (Mann-Whitney U = 208, p < 0.001).

**Fig 4:**
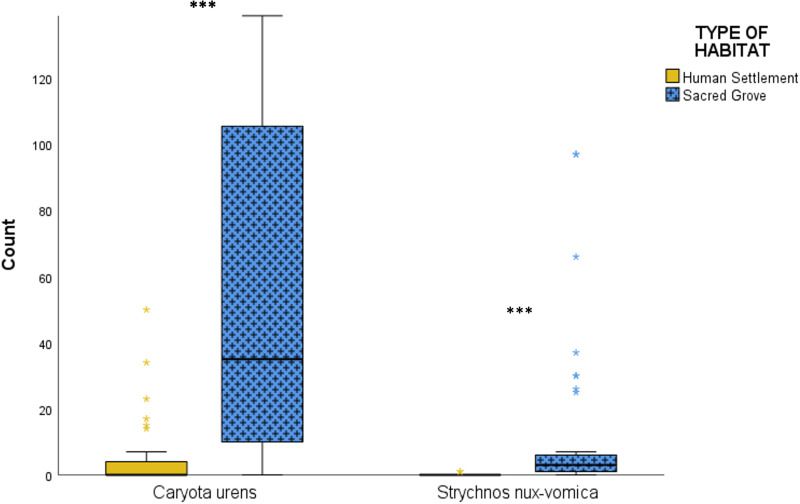
Comparison between the number of saplings of Caryota urens and Strychnos nux-vomica. The median and one quartile box plots compare the number of saplings of Caryota urens and Strychnos nux-vomica under 82 giant trees located in human settlements and sacred groves of Vanzole village, in the Northern Western Ghats. The box represents the interquartile range (IQR) with the center line denoting the median. Whiskers extend to the highest and lowest values within 1.5 times IQR. Open circles indicate mild outliers (1.5 times IQR to 3 times IQR), stars indicate extreme outliers (>3 times IQR) and asterisks indicate significance (*** p < 0.001).

One or more saplings of *S. nux-vomica* were found under 44% of all sampled trees, with a considerably higher percentage in sacred groves (77%) than in human settlements (5%). Overall, there were significantly more saplings of *S. nux-vomica* under giant trees in sacred groves (mean = 11.3, median = 3, SD = 22.8) than in human settlements (mean = 0.05, median = 0, SD = 0.23) (Mann-Whitney U = 206, p < 0.001). There were two giant trees of *S. nux-vomica* in this study, both located in sacred groves. Due to the potential for self-propagation, we removed those two individuals and reevaluated the data. The result was still significant, with significantly more saplings of *S. nux-vomica* under just the heterospecific giant trees in sacred groves (mean = 9.62, median = 2.5, SD = 21.6) than heterospecific giant trees in human settlements (mean = 0.05, median = 0, SD = 0.23) (Mann-Whitney U = 206, p < 0.001). (Fig 4).

## Discussion

India is experiencing rapid urbanization, with a growing proportion of its population residing in urban centers [52]. This demographic shift, coupled with economic pressures in rural areas, can undermine traditional conservation practices and lead to forest degradation through land conversion and logging. Within this changing landscape, remnant giant trees and sacred groves provide critical ecological refugia [9], potentially supporting multiple ecosystem services. In Vanzole Village, situated in the Western Ghats biodiversity hotspot, the local community has traditionally conserved and maintained four sacred groves. This study aimed to evaluate the contribution of giant trees towards supporting local biodiversity by addressing two key questions: 1) How do the species richness and physical dimensions of giant trees differ between sacred groves and human settlements? and 2) Does the recruitment of zoochorous saplings under giant trees differ between these two land use types?

### Biodiversity and physical structure of giant trees in sacred groves and human settlements

Regarding our first question, comparing species richness of giant trees in sacred groves and human settlements, we found that sacred groves harboured a higher species richness of giant trees (14 species) compared to human settlements (8 species). This supports existing research highlighting the floristic distinctiveness of sacred groves [25]. We also noted differences in species composition, with 12 giant tree species exclusive to sacred groves, while 6 were unique to human settlements. The dominance of *Mangifera indica* and *Artocarpus heterophyllus*, species that provide food resources, in human settlements, coupled with lower overall diversity of other species, suggests a selection bias towards economically valuable trees. The substantial differences in large tree species between sacred groves and the surrounding landscape matrix suggest that wildlife communities may utilize and perceive these habitats differently.

We also evaluated differences in the physical dimensions of giant trees in sacred groves and human settlements. We found that giant trees in human settlements exhibited larger diameter at breast height (DBH) and canopy size, whereas those in sacred groves were significantly taller. This morphological difference aligns with established ecological principles, where intense light competition in forested environments typically favours vertical growth, resulting in taller trees, while trees in more open habitats, such as human settlements and fields tend to allocate resources towards lateral expansion of the trunk and canopy [53]. These structural differences in tree morphology, particularly height, are ecologically significant as they can influence habitat selection by frugivorous species. Studies have found preferential use of tall trees by avian frugivores, potentially due to better foraging opportunities and reduced predation risk [30]. Furthermore, sacred groves have been shown to support higher avian diversity and abundance compared to random sites, with a notable increase in frugivorous guilds that frequently utilize the mid and upper canopies of tall trees [14]. Collectively, these findings suggest that the characteristic physical structure of trees in sacred groves – specifically their greater height and emergent canopies, coupled with the distinct tree species composition often found within them – may enhance their suitability for nesting, roosting, and foraging by frugivorous bird species.

### Zoochorous sapling recruitment in sacred groves and human settlements

Regarding our second question, evaluating if recruitment of zoochorous saplings differs under giant trees in sacred groves and human settlements, we observed significantly higher abundance of *Caryota urens* and *Strychnos nux-vomica* saplings in sacred groves compared to human settlements. *C.urens* saplings were found under 91% of giant trees in sacred groves versus 47% in human settlements. The presence of *C. urens* seedlings under nearly half of trees in human settlements indicates that some successful seed dispersal and initial recruitment is occurring in these more developed landscapes. Our research team has documented male Great Hornbill (*Buceros bicornis*), the Malabar Pied Hornbill (*Anthracoceros coronatus*) feeding on *Caryota urens* and transmitting the fruits to the female in the nest (Appendix A: Figure A1). However, given its moderate seed size (approximately 1 cm in diameter), dispersal of *C. urens* may not be solely attributed to large-gaped frugivores such as hornbills, but could also involve smaller avian species, such as the Asian Koel *(Eudynamys scolopaceus*), which may exhibit greater tolerance for human-modified environments [26]. Role redundancy of smaller frugivores has been shown to maintain some tree species that would otherwise be vulnerable to reductions in larger frugivore species [54].

However, the contrast was stark for *Strychnos nux-vomica*. Saplings were found under 77% of trees in sacred groves but only 5% of trees in human settlements. *S. nux-vomica* produces larger fruits (5–6 cm) which, along with various fig species, constitute a preferred food source for large-gaped frugivores, particularly hornbills during the non-breeding season in the Western Ghats [28]. This larger fruit size likely imposes constraints on consumption and subsequent dispersal by smaller avian frugivores prevalent in the wider landscape. This observation presents an interesting contrast to findings in fragmented European landscapes, where a shift towards larger and more mobile frugivore communities has been documented compared to contiguous forests [33]. In the Asian context, however, concerns exist that many large-bodied frugivores avoid human-dominated landscapes, potentially leading to limitations in effective seed dispersal within these areas [30]. While certain hornbill species, such as the Malabar Grey and Indian Grey Hornbill, may be adapting to urban and settled environments in some regions, we observed that larger species like the Great Hornbill and Malabar Pied Hornbill generally avoid human settlements unless undisturbed, large trees with suitable resources are present. This, along with potential effects of livestock grazing and other disturbances, may contribute to the much-reduced sapling recruitment of *S. nux-vomica* in the human settlements of this study.

While we did not encounter direct evidence of active hunting within our study sites, other studies have noted how hunting pressure on hornbill populations can reduce populations and seed dispersal effectiveness [32,54,55]. It is generally understood that hunting pressure on these avian frugivores varies geographically across India and appears to be less intense in our specific study region compared to the northeast region.

### Implications and limitations

Our findings suggest that while remnant trees in settlements provide some connectivity, they are not functionally equivalent to sacred groves for the recruitment of large-seeded tree species. Research has demonstrated that Great Hornbills (*Buceros bicornis*) exhibit a preference for nesting in taller trees with larger DBH that emerge significantly (by an average of 12.7 meters) above the surrounding forest canopy [5]. James and Kannan (2009) also documented Great Hornbill nests in *Terminalia bellerica* and *Bombax ceiba*, both of which were abundant and exclusively observed within the sacred groves in our study. However, individual trees within human settlements may also provide critical nesting habitat. Local villagers reported consistent annual nesting of Great Hornbills in a *Mangifera indica* within the human settlement area. As secondary cavity-nesters, Great Hornbills form monogamous pairs that often reuse the same nest sites across successive years [56]. Beyond hornbills, we also documented other species utilizing large trees for nesting, including a Crested Hawk Eagle (*Nisaetus cirrhatus*) on a *Bombax ceiba* within the Sacred Grove of Kedarling, and substantial colonies of Giant Honey Bees (*Apis dorsata*) on both *Bombax ceiba* and *Mangifera indica* within other sacred groves.

Several limitations should be considered when interpreting the findings of this study. Our small sample size precluded statistical comparisons among the four surveyed sacred groves. This could be an important area of future investigation as our qualitative observations revealed notable differences among the groves, such as reduced under-canopy vegetation in the Sacred Grove of Jugai Devi, perhaps related to cultural practices. Also, we did not document frugivorous bird visitation directly. Further research might also explore the potential contributions of other vertebrate seed dispersers, including fruit bats, macaques and other primates, and civets. It is important to note that while we documented sapling presence, which indicates successful dispersal and germination, this does not guarantee survival to maturity. In human settlements, factors such as livestock grazing, trampling, and removal of undergrowth likely reduce the survival rate of established saplings compared to the protected environment of sacred groves.

## Conclusion

Despite their often limited spatial extent (average size of 6587 m^2^ in this study), sacred groves across India represent important reservoirs of biodiversity, supporting a high richness and abundance of various taxonomic groups, including plants, animals, and other species [16,18]. Our results show that these groves act as critical reservoirs for giant, tall trees and support the recruitment of tree species that rely on large-gaped frugivores. However, sacred groves in some areas face growing anthropogenic pressures, including the construction of large cemented religious structures in lieu of sustainable alternatives, and the clearing of vegetation for development or resource extraction [25]. The documented correlation between strong religious adherence and high biodiversity in global biodiversity hotspots [57] underscores the importance of maintaining cultural and spiritual traditions, such as those associated with Hinduism, Buddhism, and animism, in maintaining biodiversity in otherwise changing landscapes.

Community-based conservation has been critical in maintaining the ecological integrity of sacred groves throughout history [58], and their significance for sustainable development is increasingly acknowledged [7,22]. Effective conservation of giant trees and sacred grove biodiversity requires engaging local communities as key stakeholders and integrating sustainable resource use with tangible economic benefits. In villages of Northern Western Ghats, Applied Environmental Research Foundation (AERF) is working to create a synergistic relationship between conservation and local livelihoods through programs such as ecological restoration of sacred groves, carbon stock estimation for potential financial incentives, and Fair Wild certification for valuable medicinal trees like *Terminalia bellirica* (Beheda). The long-term protection of giant trees and the associated biodiversity within Vanzole’s sacred groves is intrinsically linked to the successful implementation and sustained support of such community-centered conservation endeavors.

## Supporting information

S1 Fig A1. Documentation of male Malabar Pied Hornbill (*Anthracoceros coronatus*) (A, B) and Great Hornbill (*Buceros bicornis*) (C) transmitting *Caryota urens* fruits to nesting females (Photo credits: Gunwant Mahajan).
(TIFF)

S1 Data Pai etal dataset. (XLSX)
