# Supplementary figures and images for: Giant trees enhance zoochorous plant dispersal in sacred groves and human settlements of the Western Ghats, India
Source: PLoS One. 2026 May 4;21(5):e0343978. doi: 10.1371/journal.pone.0343978 (PMC13138615; doi:10.1371/journal.pone.0343978)

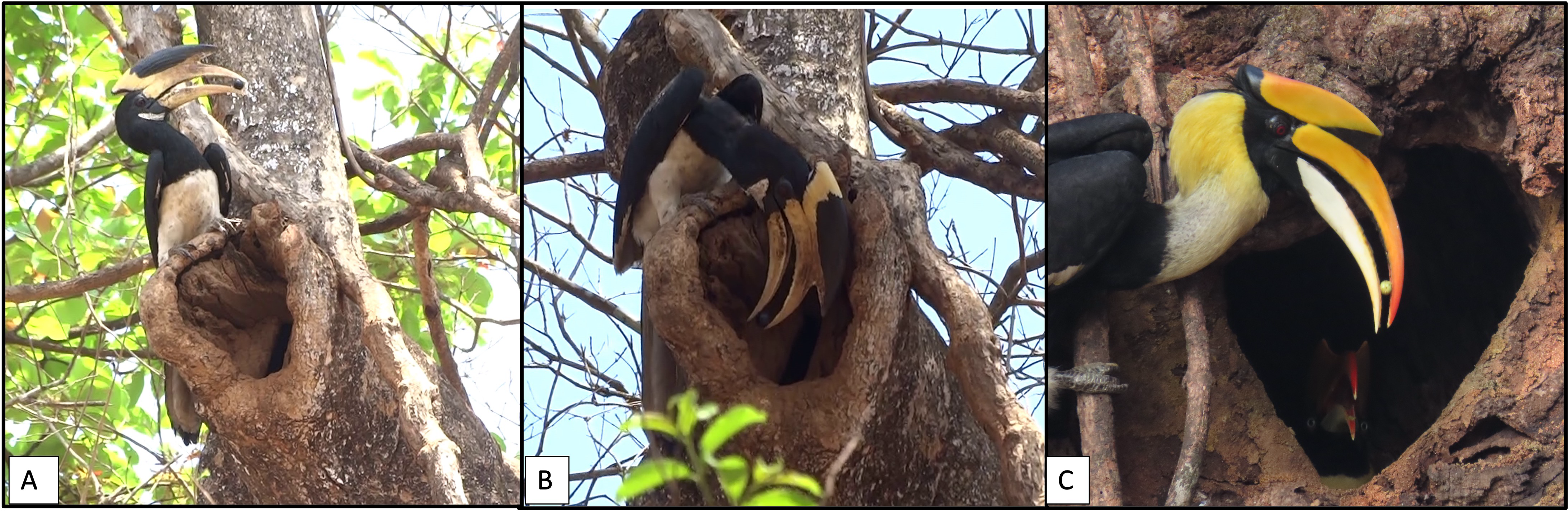

Supplement: S1 Fig — Documentation of male Malabar Pied Hornbill (Anthracoceros coronatus) (A, B) and Great Hornbill (Buceros bicornis) (C) transmitting Caryota urens fruits to nesting females (Photo credits: Gunwant Mahajan). (TIFF) [file pone.0343978.s001.tiff]
